# Neural Evidence of Cross-domain Structural Interaction between Language and Arithmetic

**DOI:** 10.1038/s41598-018-31279-8

**Published:** 2018-08-27

**Authors:** Tomoya Nakai, Kazuo Okanoya

**Affiliations:** 1 Graduate School of Arts and Sciences, The University of Tokyo, Tokyo, Japan; 2 Japan Society for the Promotion of Science, Tokyo, Japan; 3 Center for Information and Neural Networks (CiNet), National Institute of Information and Communications Technology and Osaka University, Osaka, Japan; 4 Center for Evolutionary Cognitive Science, The University of Tokyo, Tokyo, Japan

## Abstract

The presence of a shared neural system for the syntactic processing in language and arithmetic is controversial. Recent behavioral studies reported a cross-domain structural priming between language and arithmetic. Using functional magnetic resonance imaging, we examined whether the neural activation reflects the structural interaction between language and arithmetic. We prepared sentences and arithmetic expressions (A-expressions) with same and different syntactic structures and presented structurally congruent/incongruent pairs consecutively. By directly comparing activations in the congruent and incongruent conditions, we observed significant repetition suppression effect in the regions including the bilateral inferior frontal gyrus, i.e., neural activation with an A-expression decreased after a sentence with the same syntactic structure (and vice versa). The results strongly support the idea that arithmetic and language share the neural basis for processing syntactic structures.

## Introduction

Both language and arithmetic appear to be unique to human. According to the linguistic theory, the syntactic structure in natural language is based on the recursive computation of words and phrases, and the resultant structure of the recursive computation is described in the form of a hierarchical tree structure^1^. For example, a sentence such as “John ate apples” is composed first by the combination of “ate” and “apples,” and then the resultant phrase is combined with “John” to make a whole sentence. This procedure is described in the right-branching form [John [ate apples]], or equivalently in the form of tree structure.

**Figure 1:**
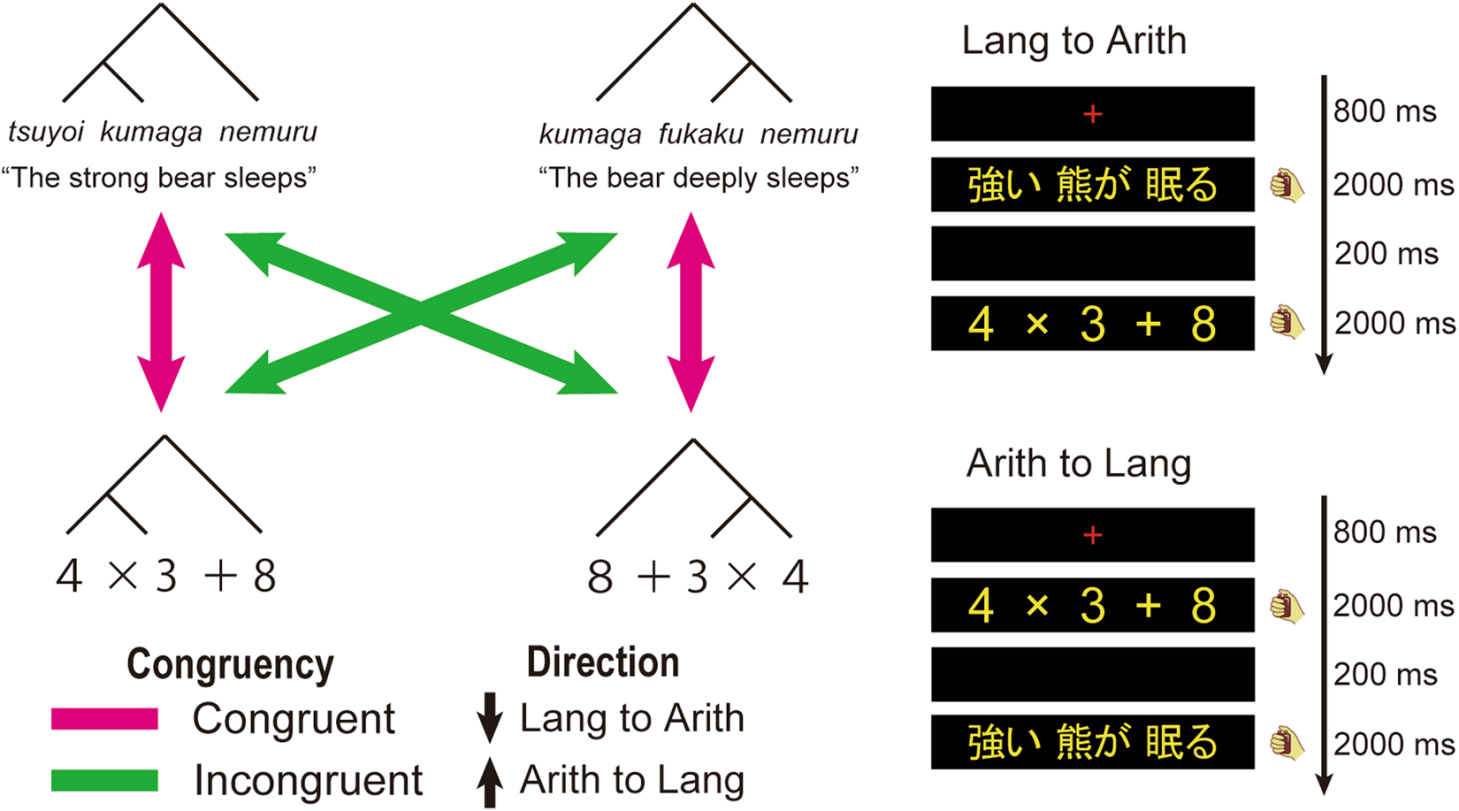
Illustration of the task design. (**A**) Example stimuli of language (Lang) and arithmetic (Arith) tasks in the RS sessions are shown. Japanese sentence stimuli (denoted in italics) and their English translation are depicted together. Cyan and green arrows indicate congruent and incongruent conditions, respectively. (**B**) Time course of stimuli presentation is shown. Stimuli are presented either in Lang to Arith (top) or in Arith to Lang direction (bottom).

It has been argued that recursion provides a basis not only for syntactic processing in language but also for other symbolic systems such as arithmetic expressions (A-expressions)^2^. One theoretical approach has associated tree structures to the basic A-expressions based on the order-rule of arithmetic operations^3^. For an A-expression such as “3 × 4 + 8”, the sub-expression “3 × 4” is recursively combined with “8” by addition. This procedure is described in the left-branching form [[3 × 4] + 8], which is equivalent to the tree structure description (Fig. 1). It is therefore a critical question whether the tree structure descriptions of language and arithmetic merely reflect the superficial similarities of language and arithmetic, or are based on the common neural systems for recursion.

Behavioral studies have indicated that processing of A-expressions is likely based on the syntactic structures^4,5^. For example, participants moved their eyes along with syntactic structures of sentences^6^. A recent behavioral study has reported that syntactic structures of prime A-expressions affected processing of following target sentences^7^. A subsequent study has revealed the bidirectional syntactic priming effect (i.e., both from language to arithmetic [Lang to Arith] and from arithmetic to language [Arith to Lang])^8^. These reports indicate the existence of a shared syntactic basis for language and arithmetic, which may induce the cross-domain syntactic interaction between the two domains.

Based on the large number of studies on syntactic processing in language^9–15^, the left inferior frontal gyrus (IFG), traditionally called Broca’s area, has been considered as a core region for the syntactic processing in language. The opercular part of the left IFG was more activated in the syntactic judgment task compared to the semantic judgment^10^. It has been further reported that activation in the left IFG was modulated by the hierarchical levels of syntactic structures for the normal/pseudowords sentences^14^, indicating that the left IFG reflects structural property of the language syntax.

Other regions, such as the anterior temporal lobe (ATL) and superior temporal gyrus (STG) have also been suggested to be involved in the syntactic processing^14–19^. The left ATL and left STG are often coactivated with the IFG^14,15^, while several studies have indicated distinct activation patterns. For example, syntactic processing during natural story listening induced activation in the left ATL rather than the left IFG^18^. These regions would be also candidates for syntactic (i.e., structural) processing in language.

Whether there is a shared neural basis for language and arithmetic is still under debate. Several neuroimaging studies have reported very small or no activations in language-related regions for arithmetic tasks^20–24^. Even patients with large left-hemisphere perisylvian lesions and severe grammatical impairment showed various numerical capacities^25,26^. In contrast, other functional Magnetic Resonance Imaging (fMRI) studies showed shared activation for syntactic processing in language and arithmetic in the left IFG^27,28^. A lesion study with a large number of patients has also suggested a joint function of the left IFG for language and arithmetic^29^. The overlap of fMRI activation in a certain region, however, does not imply that the two domains are based on the *same* system. To test this possibility, the repetition suppression (RS) effect is useful. The RS effect refers to the attenuation of neural activation in a certain neural circuit with repeated exposure to certain stimuli. The RS effect based on syntactic structures has repeatedly been observed in the language domain^30–32^. Therefore, the existence of a cross-domain structural RS effect between language and arithmetic would support the idea of shared neural system for language and arithmetic. The goal of the current study is thus to demonstrate the cross-domain structural RS effect between language and arithmetic, especially in the syntax-related region such as the left IFG.

Previous studies have shown cross-domain structural interaction only in behavioral data^7,8^, and the neural basis of structural interaction has been unknown. Although neural structural RS effects have been observed for the *intra-domain* language stimuli^30–32^, no study has ever reported *cross-domain* structural RS effect between language and arithmetic in the brain. Here, we used a cross-domain structural priming paradigm between language and arithmetic (Fig. 1). For both A-expressions and sentences, we prepared left-branching (LB) and right-branching (RB) stimuli, thus generating structurally congruent and incongruent conditions. In a 3-T MRI scanner, 20 participants were consecutively presented with a sentence and A-expression (or vice-versa), and they performed semantic judgment task/calculation task during four sessions. We adopted this experimental setting because previous studies have reported the structural RS effect in language within a span of single trial^30–32^. By directly comparing the neural activations in the congruent and incongruent conditions in both directions (i.e., Lang to Arith and Arith to Lang), we investigated the brain regions that reflected cross-domain structural interactions. Among multiple regions related to language and arithmetic, we especially focused on the function of the bilateral IFG, because the contribution of the bilateral IFG for syntactic processing in language and arithmetic has been a central question in numerous previous studies^20–29^. The current study is the first report of neural evidence of cross-domain structural interaction between language and arithmetic.

## Materials and Methods

### Participants

Twenty college students (ages 19–22, all male) were recruited for this study. All participants were healthy native Japanese speakers and right-handed (laterality quotient, LQ: 66.7–100), as determined by the Edinburgh inventory^33^. We recruited participants majoring in natural sciences, because a previous study has reported larger cross-domain syntactic priming effect for the participants in natural science discipline^7^. Prior to their participation in the study, written informed consent was obtained from all participants. The experiment was approved by the Ethics Committee of the University of Tokyo, Komaba. All methods were performed in accordance with the relevant guidelines and regulations.

### Stimuli

For both A-expressions and sentences, we composed 16 left-branching (LB) and 16 right-branching (RB) stimuli. LB sentences were composed of an adjective, noun, and intransitive verb (e.g., *Tsuyoi kumaga nemuru*, the strong bear sleeps), whereas RB sentences were composed of a noun, adverb, and intransitive verb (e.g., *Kumaga fukaku nemuru*, the bear deeply sleeps). Semantically normal sentences contained a pair of combinable noun and verb. In contrast, semantically anomalous sentences contained a pair of incombinable noun and verb (e.g., *Tsuyoi kumaga tsumoru*, the strong bear accumulates). A-expressions were composed of three single digits and two operators (addition and multiplication), without any parentheses. LB A-expressions had multiplication embedded into the left part (e.g., 3 × 4 + 8), while RB A-expressions had multiplication embedded into the right part (e.g., 8 + 4 × 3). The calculation results of the standard A-expressions were always 20 or 30 (i.e., multiples of 10). Deviant A-expressions always had results of +1 or −1 to the standard expressions (e.g., 3 × 4 + 7).

All stimuli pairs were divided into two conditions (congruent and incongruent). In the congruent condition, LB was presented after LB, or RB was presented after RB. In the incongruent condition, LB was presented after RB, or RB was presented after LB. Half of the stimuli were semantically normal or standard stimuli, while the other half were anomalous or deviant. Standard and Deviant stimuli pairs were equally included in both congruent and incongruent pairs.

### Procedure

At the beginning of each trial, a small red cross was shown for 800 ms at the center of the screen. In the first four sessions (RS sessions), in each trial, an A-expression and sentence were consecutively presented each for 2000 ms, with a 200-ms blank between two stimuli. The presentation order was either an A-expression after a sentence (Lang to Arith) or vice-versa (Arith to Lang). The inter-trial interval (ITI) was 12000–15000 ms. We also performed two functional localizer sessions, where, in each trial, an A-expression or sentence was individually presented for 2000 ms after the presentation of a small red cross for 800 ms. The ITI of the localizer sessions was 4500–7500 ms.

Each of the RS sessions consisted of 16 trials (four trials under each condition for both Lang to Arith and Arith to Lang directions), and each of the subsequent two localizer sessions consisted of 32 trials. The order of trials was randomized and counter-balanced across participants. Each additional session consists of four blocks, where each block contained eight trials composed of one of the four stimulus types (i.e., LB/RB sentences and A-expressions), separated by a 15-s Rest period.

The participants were asked to perform the task during the presentation of each A-expression/sentence and to respond as soon as possible. For the A-expressions, the participants were asked to judge whether the result of a calculation was a multiple of 10 by pressing either the left or right button. For the sentences, the participants were asked to judge whether the sentence was natural or not. To control the effect of hand use, we asked the half of the participants to use the right hand for the button response, while we asked the other half to use the left hand.

The participants wore earplugs in the scanner. Stimuli were presented on a liquid-crystal display monitor (resolution: 1920 × 1080) so that the participants viewed them through a mirror. Reaction time (RT) was measured from the onset of each sentence/expression stimulus. Participants were also asked to rate the acceptability of all linguistic stimuli on a 5-point Likert scale from 1 (*totally unacceptable*) to 5 (*perfectly acceptable*). The stimulus presentation and collection of behavioral data were controlled using the Presentation software (Neurobehavioral Systems, Albany, CA). After the MRI experiment, we verbally asked participants whether they recognized structural relationship (i.e., the left-branching and right-branching combination) of the stimuli. None of the participants reported the recognition of the structural relationship between two stimuli.

### MRI Data Acquisition

The functional imaging was conducted on a 3.0 T scanner (MAGNETOM Prisma; Siemens, Erlangen, Germany) with a 64-channel head coil. We scanned 68 interleaved axial slices that were 2.0-mm thick without gap, parallel to the anterior and posterior commissure line, using a T2*-weighted gradient-echo multiband echo-planar imaging (MB-EPI) sequence^34^ [repetition time (TR) = 1500 ms, echo time (TE) = 30 ms, flip angle (FA) = 60°, field of view (FOV) = 192 × 192 mm^2^, resolution = 2 × 2 mm^2^, MB factor = 4]. We obtained 140 volumes in the RS sessions and 168 volumes in the additional sessions, each following four dummy images, which allowed for the rise of the MR signals. For anatomical reference, high-resolution T1-weighted images of the whole brain (192 sagittal slices, 1 × 1 × 1 mm^3^) were also acquired from all participants with a Magnetization Prepared Rapid Acquisition Gradient Echo sequence (TR = 1900 ms, TE = 2.0 ms, FA = 9°, FOV = 240 × 256 mm^2^).

### Data Exclusion

Correct/incorrect trials were evaluated by the response matching regardless of RTs, and trials with no response were treated as incorrect trials. We excluded incorrect trials from RT analysis. We further excluded trials with RT more than mean + twice the standard deviation, or less than mean – twice the standard deviation as outliers. By the above procedure, on average 5.2% of the prime stimuli and 3.8% of the target stimuli were excluded. We excluded the incorrect trials also from fMRI data analyses.

### fMRI Data Analysis

We performed fMRI data analyses using SPM12 statistical parametric mapping software (Wellcome Trust Centre for Neuroimaging, London, UK; http://www.fil.ion.ucl.ac.uk/spm/). The acquisition timing of each slice was corrected using the middle slice as a reference. We realigned the EPI data from multiple sessions to the mean image across all sessions. Each participant’s T1-weighted structural image was coregistered to the mean functional image and then spatially normalized to the Montreal Neurological Institute (MNI) space. After the spatial normalization, the resultant deformation field was applied to the realigned functional images, resampled into 2 mm isotropic voxels, and then smoothed using an isotropic Gaussian kernel of 8 mm full-width at half maximum. Low-frequency noise was removed by high-pass filtering at 1/128 Hz.

We used regressors composed of the hemodynamic response function (HRF), convolved with box-car function starting from the onset of the prime stimuli (lasting until the response in the target stimuli) in the RS sessions. For the localizer sessions, we used a regressor of the HRF convolved with box-car function (duration equals to RTs). For the RS sessions, we made four regressors of congruent/incongruent conditions for both Lang to Arith and Arith to Lang directions. For the localizer sessions, we made regressors of sentences and A-expressions (the LB and RB stimuli combined), composed of the HRF starting from the onset of each stimulus. We added head motion parameters calculated during realignment process as regressors of non-interest. The statistical threshold was set at *p* < 0.05 for the voxel level, with a family-wise error (FWE) correction for multiple comparisons] across the whole brain. For the contrast which did not show significant activation with FWE correction, we used the statistical threshold of uncorrected *p* < 0.005 and *k* > 10 as suggested elsewhere^35^.

For the region of interest (ROI)-based beta estimate analysis, we defined two anatomical ROIs for the bilateral opercular part of the IFG with the Automatic Anatomical Labeling atlas^36^, because these regions have been a crucial target of common syntactic processing unit for language and arithmetic^20–29^. We also examined anatomical ROIs of the bilateral inferior parietal lobule (IPL), which has been reported in several studies on syntactic processing in arithmetic^27,28^. We extracted average activation across all voxels in the target ROIs.

We further prepared two additional general linear models. In the first model, we divided each of the four regressors in the RS sessions into two separate pairs which contained either semantically correct or deviant sentences. In the second model, we divided each regressor into two separate pairs which contained either LB or RB sentences. For the latter model, data from one subject was excluded because of missing conditions by incorrect trials exclusion.

## Results

### Behavioral results

According to the acceptability rating of participants, the normal sentences were classified as acceptable (mean acceptability scores and standard error of mean (SEM): 4.83 ± 0.04), while the anomalous sentences were classified as unacceptable (mean acceptability scores and SEM: 1.17 ± 0.05). Using a paired *t*-test, we found a significant difference of acceptability scores between normal and anomalous sentences [*t*(19) = 55.16, *p* < 0.001, *d* = 18.7]. Therefore, the semantic decision task of Japanese sentences seemed to be well-established and reliable.

**Figure 2:**
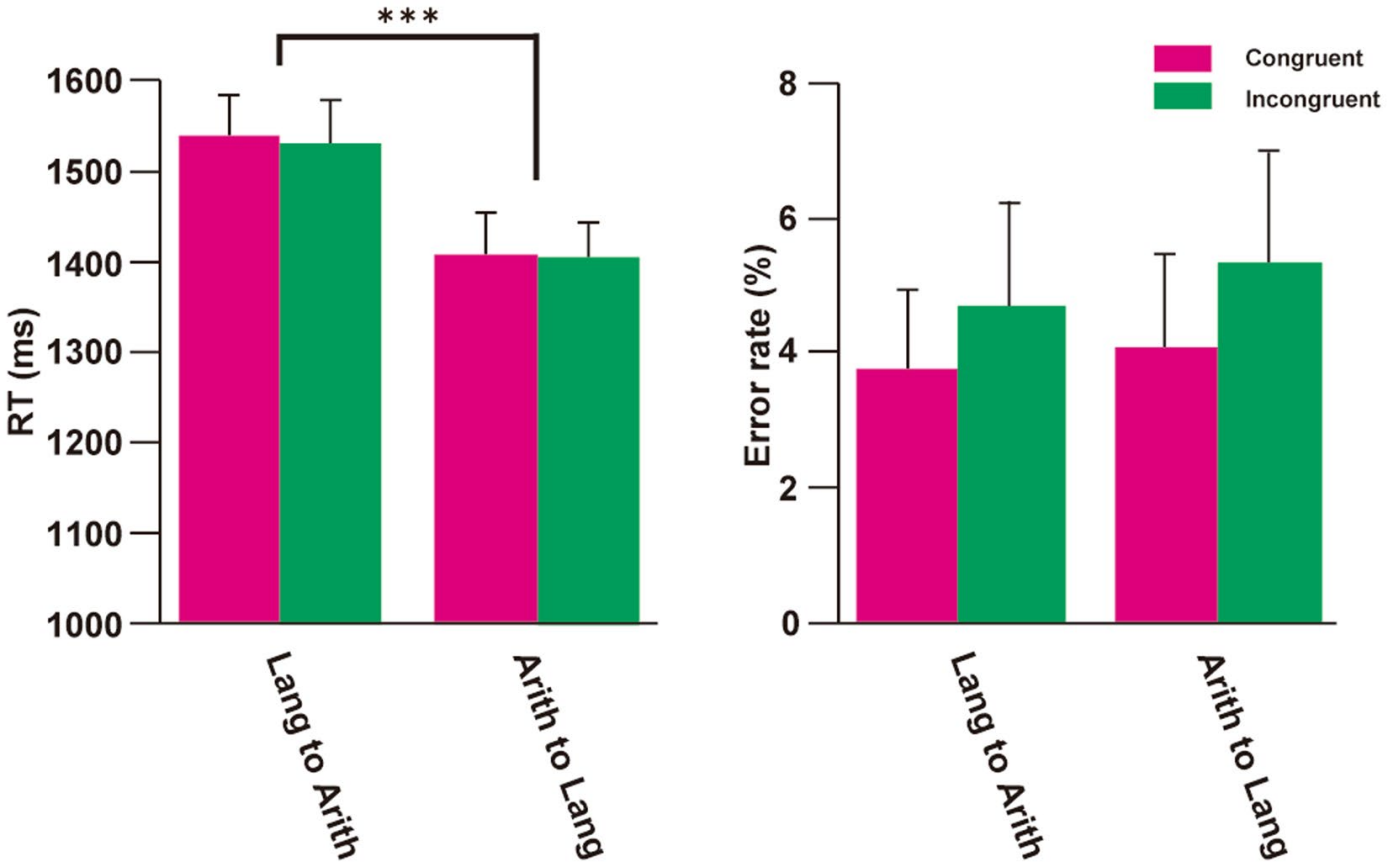
Behavioral results in the RS sessions. RT and error rates of the target stimuli in the RS sessions are displayed. Congruent condition is marked in cyan, while Incongruent condition is marked in green. Data in both Lang to Arith and Arith to Lang directions are shown. ***p < 0.001. Error bars, SEM.

To examine whether there is a structural priming effect between language and arithmetic, we analyzed behavioral measures of target stimuli (Fig. 2). A two-way analysis of variance (rANOVA) of the RT in the target stimuli did not show a significant main effect of Congruency (Congruent vs. Incongruent), but only the main effect of Direction (Lang to Arith vs. Arith to Lang) [*F*(1,19) = 15.99, *p* = 0.00077, *η*_*p*_^2^ = 0.84]. Post-hoc *t*-tests (*α* = 0.013, Bonferroni correction) showed longer RT for the Lang to Arith direction compared to the Arith to Lang direction for both conditions (*p* < 0.0014). We also examined behavioral measures in the localizer sessions (see Supplementary Fig. S1). Neither RTs nor error rates showed significant difference between Lang and Arith tasks (RTs, *p* = 0.17; Error rates, *p* = 0.45), indicating that task demands were comparable between Lang and Arith tasks.

### Cross-domain structural RS effect

**Figure 3:**
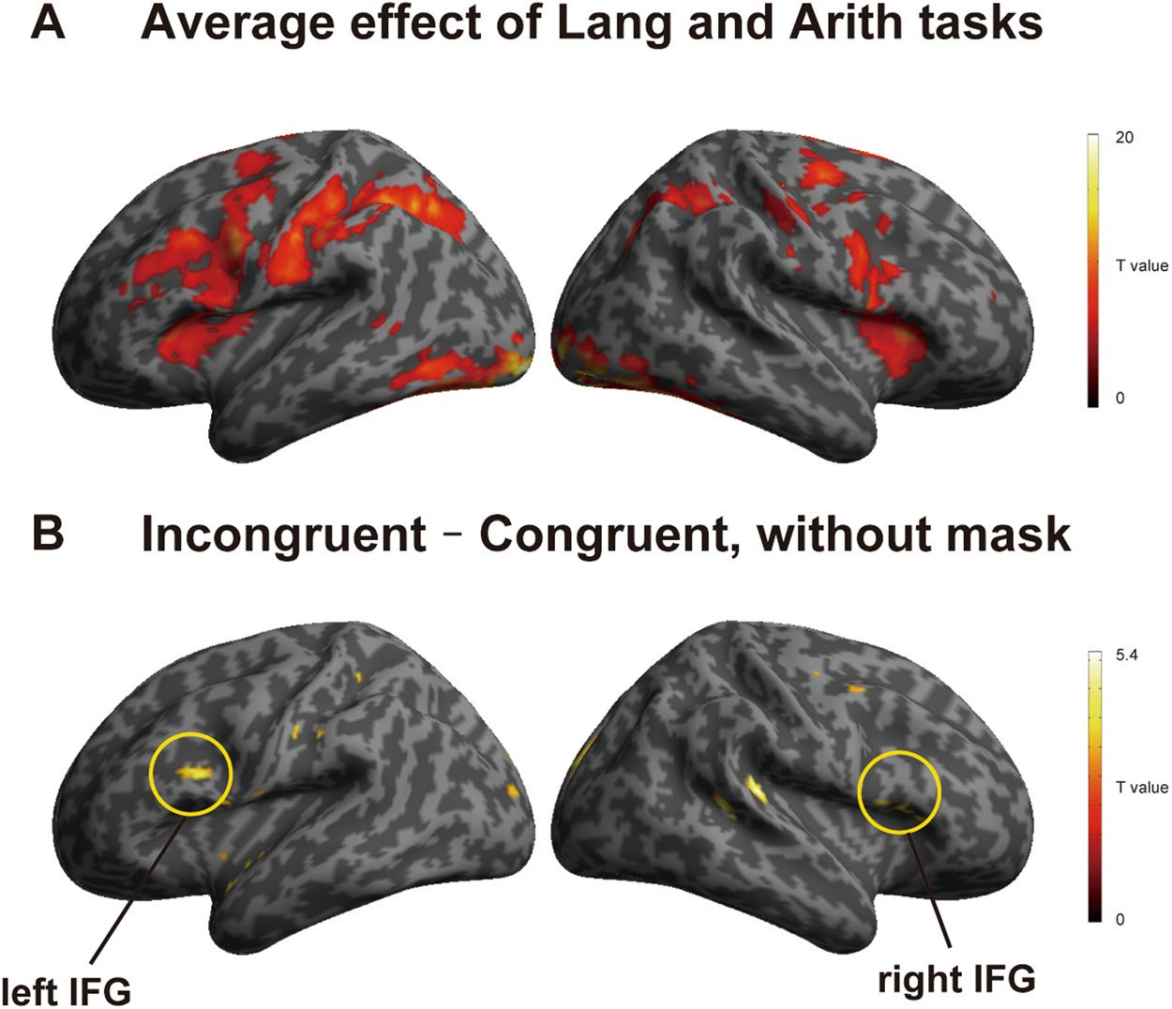
The Incongruent – Congruent contrast without inclusive masking. (**A**) Average effect of Arith and Lang, which were independently performed in the localizer sessions (*p* < 0.05, FWE corrected). (**B**) The cortical activation map of Incongruent – Congruent contrast without inclusive masking (*p* < 0.005, *k* > 10).

By analyzing fMRI data of the functional localizer sessions, we found activation in large brain regions including the bilateral IFG common to Lang and Arith tasks (voxel *p* < 0.05, FWE correction, Fig. 3A). The functional localizer image was later used as a mask image. The direct comparison between Lang and Arith tasks revealed larger activation for the Lang task in the triangular part of the left IFG, left STG, and left fusiform gyrus, as well as larger activations for Arith task in the opercular part of the bilateral IFG and bilateral inferior parietal lobule (IPL) (see Supplementary Fig. S2).

**Figure 4:**
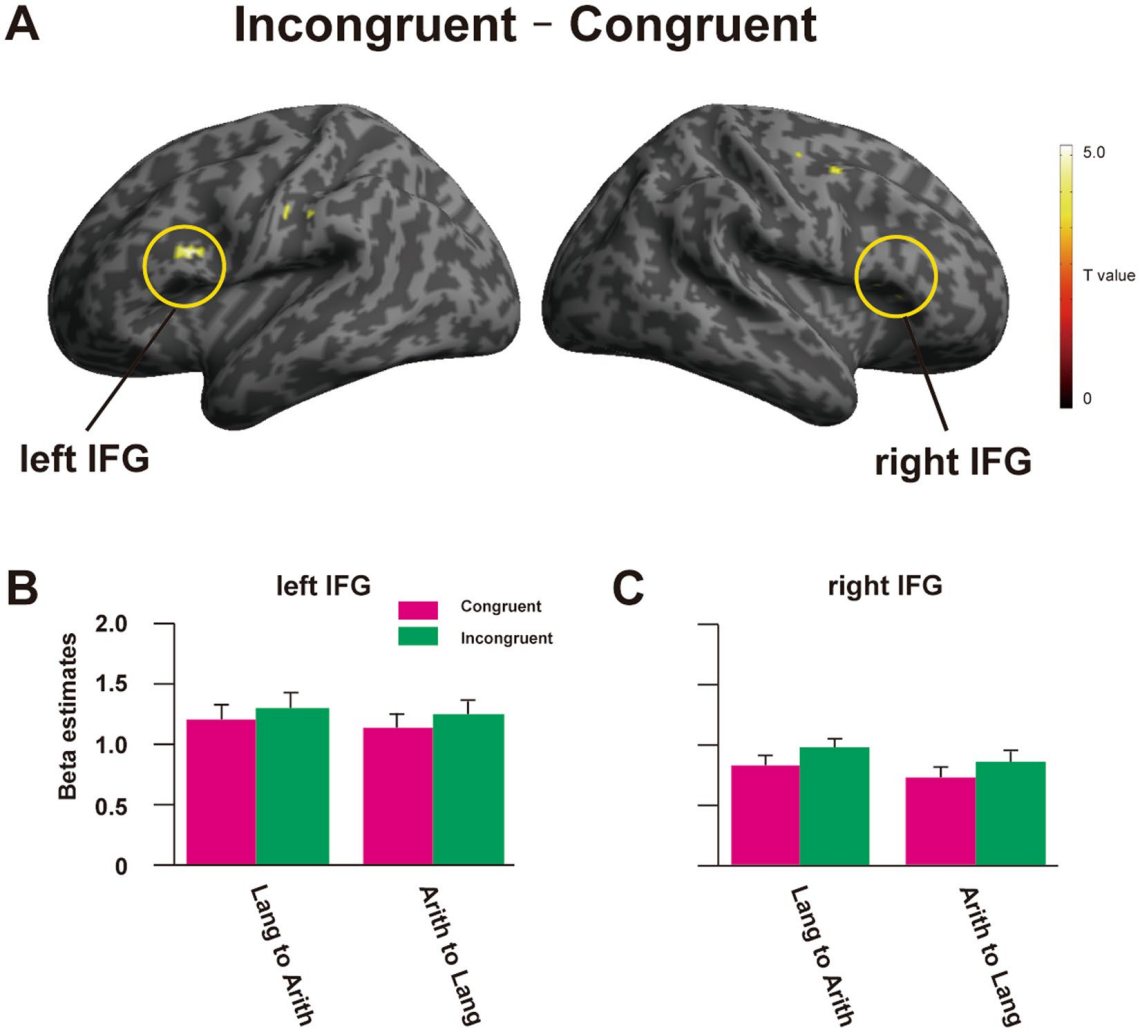
Incongruent – Congruent contrast, with inclusive masking. (**A**) The cortical activation map of Incongruent – Congruent contrast, averaged across Lang to Arith and Arith to Lang directions (*p* < 0.005, *k* > 10). Activation was masked with the additional session data where Arith and Lang tasks were independently performed. (**B**,**C**) Beta estimates for Congruent (cyan) and Incongruent (green) conditions were extracted from regions of interest (ROIs) of a 6-mm sphere from the peak voxel of the opercular part of the left IFG and right IFG, for both Lang to Arith and Arith to Lang directions. Error bars, SEM.

Using the RS session data, we examined a contrast between Incongruent and Congruent pairs for both Lang to Arith and Arith to Lang directions. We observed significant activation in the opercular part of the bilateral IFG (*p* < 0.005, *k* > 10, Fig. 3B), as well as in the left supplementary motor are (SMA), right dorsolateral premotor cortex (dLPMC), left middle frontal gyrus (MFG), left ATL, left supramarginal gyrus (SMG), right STG, left thalamus, and cerebellum. To confirm that the activated regions are involved in the language and arithmetic processing, we used the average effect of Lang and Arith tasks during the localizer sessions as an inclusive mask (Fig. 3A), and still found the RS effect in the bilateral IFG, SMA, right dLPMC, left MFG, left SMG, and left thalamus (Fig. 4A, Table 1). Importantly, the RS effect was bidirectional, i.e., in both Lang to Arith and Arith to Lang directions, as revealed by the signal changes extracted from the 6-mm sphere around the peak voxel of the bilateral IFG (Fig. 4B,C).

**Table 1:** Regions that showed activation in the Incongruent vs. congruent contrast.

Brain region	Side	*x*	*y*	*z*	*Z*-value	Voxels
SMA	M	−4	16	54	3.0	19
dLPMC	R	40	2	54	2.9	31
MFG	L	−50	36	22	3.4	17
IFG	R	44	20	4	3.3	29
	L	−46	12	22	3.3	73
SMG	L	−54	−24	32	3.3	28
Thalamus	M	−6	−18	10	4.0	64

Stereotactic coordinates (*x*, *y*, *z*) in the Montreal Neurological Institute (MNI) space (mm) are shown for each activation peak of *Z*-values. MFG, middle frontal gyrus; IFG, inferior frontal gyrus; SMA, supplementary motor area; dLPMC, dorsolateral premotor cortex; SMG, supramarginal gyrus; L, left hemisphere; R, right hemisphere; M, medial.

**Figure 5:**
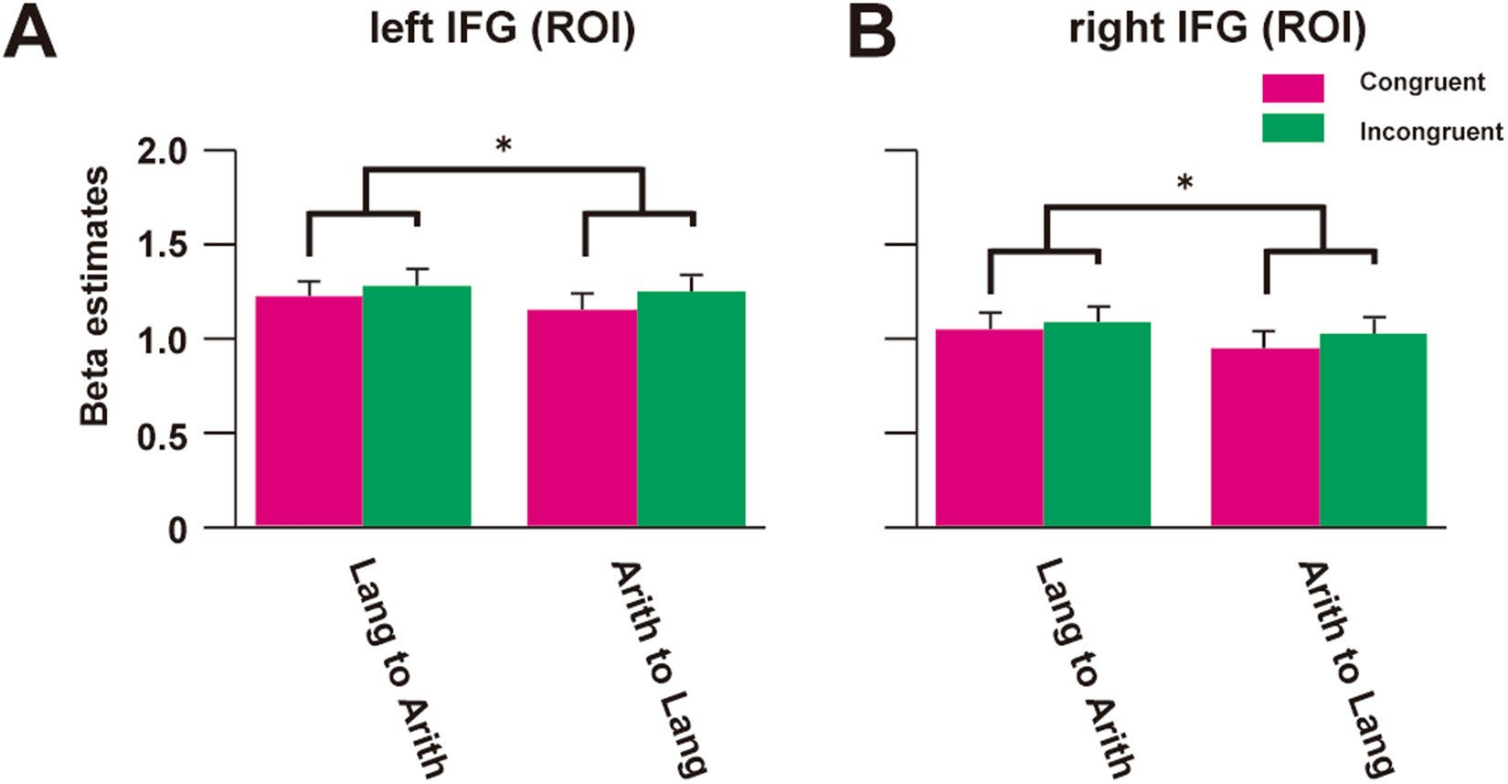
Beta estimates for Congruent and Incongruent conditions. Beta estimates for Congruent (cyan) and Incongruent (green) conditions were extracted from the independent anatomical ROIs of (**A**) the left IFG and (**B**) right IFG, for both Lang to Arith and Arith to Lang directions. *p < 0.05 for the main effect of Congruency. Error bars, SEM.

Among multiple candidate regions for syntactic processing in language and arithmetic, the bilateral IFG showed RS effect in the whole-brain analyses. To confirm that our results of RS effect in the IFG are robust, we extracted signals from the independent anatomical ROIs (Fig. 5). The two-way rANOVA in the left IFG showed a significant main effect of Congruency [*F*(1,19) = 8.74, *p* = 0.022, *η*_*p*_^2^ = 0.32]. In the right IFG, the main effect of Congruency was marginally significant [*F*(1,19) = 4.42, *p* = 0.049, *η*_*p*_^2^ = 0.19].

To examine whether we find an RS effect in the arithmetic-related regions, we performed additional ROI analyses in the bilateral IPL (see Supplementary Fig. S3). The bilateral IPL did not show a significant RS effect, but a significant main effect of Direction [left IPL, *F*(1,19) = 10.26, *p* = 0.0047, *η*_*p*_^2^ = 0.35; right IPL, *F*(1,19) = 8.15, *p* = 0.010, *η*_*p*_^2^ = 0.30].

To exclude the effect of semantic appropriateness of language task, we compared RS effects (i.e., differences between incongruent and congruent conditions) in normal and deviant sentences. We found no significant difference of RS effects in the left and right IFG (*p* > 0.22) (see Supplementary Fig. S4). Finally, we tested the effect of branching structures (LB/RB) of language task. The RS effect was larger for the pairs with LB sentences only in the right IFG (*p* = 0.025) (see Supplementary Fig. S4).

## Discussion

In the current study, we prepared structurally congruent and incongruent sentences and A-expressions, and examined the cross-domain structural RS effect using fMRI. The results showed the RS effect for both Lang to Arith and Arith to Lang directions in brain regions including the bilateral IFG.

Previous neuroimaging studies have revealed the critical involvement of the left IFG in the syntactic processing of language^9–15^. In contrast, the involvement of the left IFG in arithmetic syntax has been debated^20–29^. We showed that syntactic RS effect between language and arithmetic recruited the bilateral IFG. Anatomical ROI analysis further indicates that the RS effect was robust particularly in the left IFG, with large effect size according to the Cohen’s criteria^37^. Activation overlap leaves the possibility that two different systems exist in the same brain region. Since the RS effect refers to the attenuation of neural activation in neural circuits with repeated exposure to certain stimuli, the structural RS effect between language and arithmetic suggests that the structural aspects of the two cognitive domains are based on the same system.

We also considered the RS effect in several other candidate regions which have been associated with syntactic processing in language^14–19^. Although the left ATL and right STG showed the RS effect without masking, activation was not significant in these regions with inclusive masking of localizer session data. The absence of RS effect with masking may derive from the activation absence in these regions in the Arith task, as shown in the Arith – Lang contrast. Previous studies using arithmetic tasks have often reported activation in the fronto-parietal regions, but not in the left ATL or right STG^38^. It is therefore improbable that the left ATL and right STG are common syntactic ground for both language and arithmetic.

Although we have focused on the joint activation across language and arithmetic, the direct contrast between language and arithmetic tasks also showed inter-domain variation of activation patterns. Language task induced larger activation in the triangular part of the left IFG, while arithmetic task induced larger activation in the opercular part of the bilateral IFG. Since interaction was not significant in the series of rANOVA analyses, the RS effect itself was not dependent on the presentation direction. It is likely that the larger activations in the Lang to Arith direction demonstrated in the bilateral IPL ROI reflect activation differences between language and arithmetic tasks.

Arithmetic tasks often induce activation in the bilateral IPL^39,40^. IPL is sensitive to the magnitude changes of presented dot patterns^41,42^, as well as number words^43^. Number-selective neural circuit has been reported in the right IPL^44^, which is in line with the numerosity-sensitive neurons found in the parietal and frontal areas in primates^45,46^. Although these previous studies have suggested that the IPL is related to processing of magnitude or numerosity of presented stimuli, it has been also observed that the functional/anatomical network between left IFG and IPL is important for arithmetic performance^47,48^. It is likely that IFG is involved in arithmetic or more complex mathematical tasks rather than elementary number processing^28,49,50^. The current RS effect in the IFG suggests that structural aspect of arithmetic is beyond the processing of numerosity or magnitude.

In contrast to the previous studies of behavioral structural priming experiments^7,8,51^, we did not find significant congruency effect in RTs or error rates. Although we observed a small tendency of larger error rate in the incongruent condition than congruent condition (mean, +0.8% in Arith to Lang, +1.2% in Lang to Arith), the lack of significant effect may due to a ceiling effect (>90% of accuracy) compared to the previous behavioral study where mean accuracy was 35–79%^8^. Another possible reason is the difference in the experimental situation between the previous behavioral experiments (on questionnaire sheets) and current fMRI experiment (button pressing in the MRI scanner, with noisy scanning sound); the latter might have an influence on the participants’ attention in the tasks. There is a widely-accepted assumption that the RS effect is accompanied by the behavioral priming effect; however, RS effects without any behavioral priming effects have also been reported^52,53^. Conversely, fMRI may be a more sensitive measure than behavioral experiments for detecting the interactions of higher cognitive functions.

It is important to consider effects of other non-structural factors for the RS effect in the IFG. There is a category difference between LB and RB sentences ([[Adjective Noun] Verb] and [Noun [Adverb Verb]], respectively), which is in contrast to the symmetrical combination in arithmetic ([[Number × Number] + Number] and [Number + [Number × Number]]). In the right IFG, we found a larger RS effect for pairs with the LB sentences than those with the RB sentences, which may derive from a possible structural ambiguity of the [Adverb Verb] component in the RB sentences. Although we used manner adverbs which have been considered to be attached to the verb^54^, inherent order variability of adverbs might reduce the clarity of structural information. The contribution of other linguistic component such as semantics and phonology, as well as that of the general cognitive factors such as working memory or task difficulty, is minimized in the comparison between Incongruent and Congruent conditions, because those two conditions consist of the same set of sentences/A-expressions. The only difference between Incongruent and Congruent conditions is the structural combination of the prime and target stimuli. An effect of semantic appropriateness which may exist in language task was also excluded in the additional analysis, where no significant difference was found between semantically normal and deviant sentences. In the current study we have focused on the structural aspect of language and arithmetic. Thereby we have tested three different models to segregate the effect of the structural congruency from other parameters. Further research is necessary to fully clarify the relationship between language and arithmetic including all related parameters in a single paradigm.

## Conclusion

Based on the hypothesis that language and arithmetic share the neural basis, we examined whether the neural activation reflects the structural RS effect between language and arithmetic. The observed RS effect in the region including the left IFG indicates that, the activation overlap of language and arithmetic is based on the same system for processing syntactic structures.

## Electronic supplementary material

Supplementary Information
