# Supplementary material for: Neural Evidence of Cross-domain Structural Interaction between Language and Arithmetic
Source: Sci Rep. 2018 Aug 27;8:12873. doi: 10.1038/s41598-018-31279-8 (PMC6110712; doi:10.1038/s41598-018-31279-8)
Supplement: Supplementary file 1 — Supplementary Information [file 41598_2018_31279_MOESM1_ESM.docx]

**Neural Evidence of Cross-domain Structural Interaction between Language and Arithmetic**

**Tomoya Nakai, Kazuo Okanoya**

**Supplementary Information**


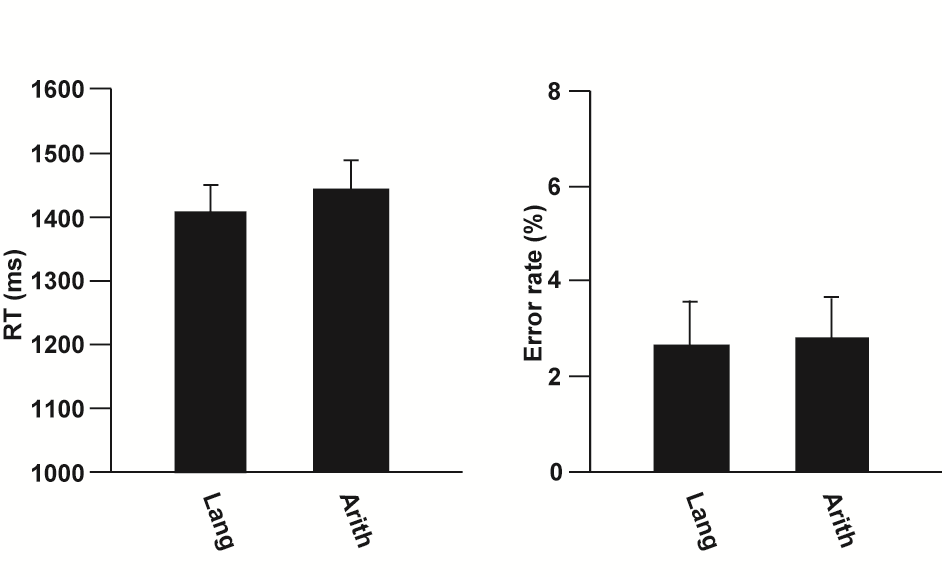


**Figure S1.** Behavioral results in the localizer sessions.

RT and error rates in the localizer sessions are displayed. Error bars, SEM.


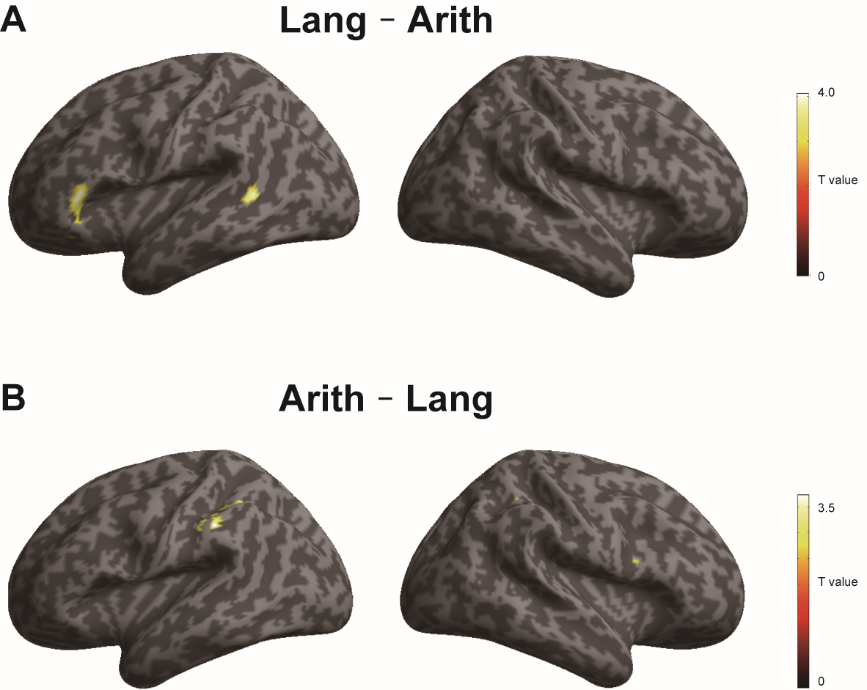


**Figure S2.** Direct comparison of Lang and Arith tasks.

The cortical activation map of (A) Lang – Arith and (B) Arith – Lang contrasts (*p* < 0.005, *k* >10). Data from localizer sessions were used.


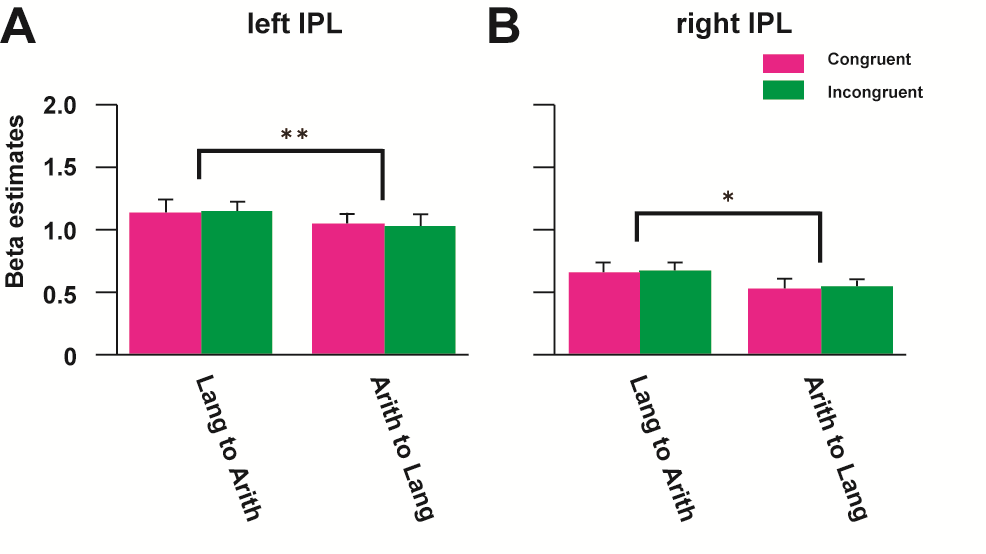


**Figure S3.** Beta estimates in the bilateral IPL.

Beta estimates for Congruent (cyan) and Incongruent (green) conditions were extracted from the independent anatomical ROIs of (A) the left IPL and (B) right IPL, for both Lang to Arith and Arith to Lang directions. **p* < 0.05, ***p* < 0.01. Error bars, SEM.


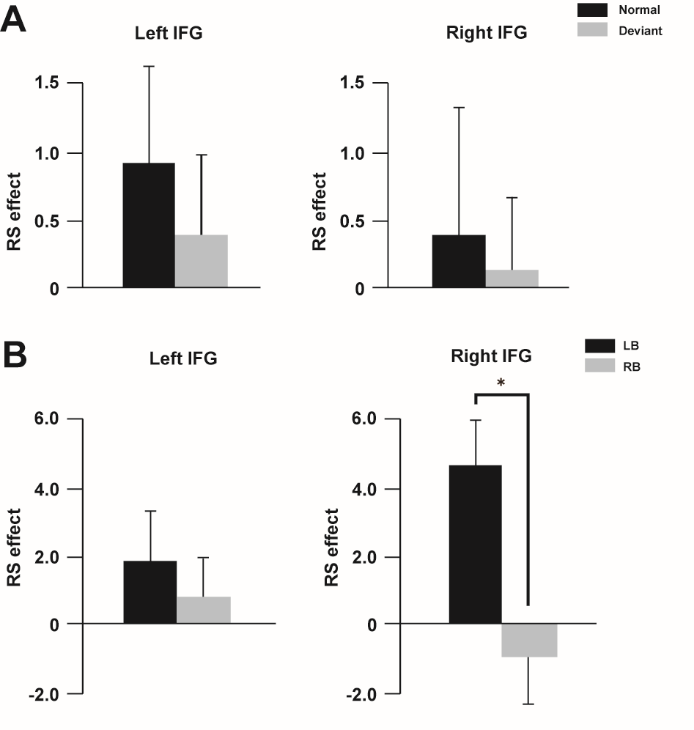


**Figure S4.** RS effect in additional analyses. RS effect size was assessed by subtracting beta estimates of the Congruent condition from that of the Incongruent condition. Data from Lang to Arith and Arith to Lang directions were averaged. (A) RS effects were separately calculated in semantically normal sentences (black) and deviant sentences (grey). (B) RS effects were separately calculated in LB sentences (black) and RB sentences (grey). **p* < 0.05. Error bars, SEM.
